# Supplementary material for: HIVprotI: an integrated web based platform for prediction and design of HIV proteins inhibitors
Source: J Cheminform. 2018 Mar 9;10:12. doi: 10.1186/s13321-018-0266-y (PMC5845081; doi:10.1186/s13321-018-0266-y)
Supplement: Supplementary file 2 — Additional file 2. Source code of HIVProtI web server. [file 13321_2018_266_MOESM2_ESM.zip › HIVprotI_Source-code/hivprotI/weka-3-6-6/doc/weka/datagenerators/classifiers/classification/package-summary.html]

weka.datagenerators.classifiers.classification


---


|  |  |  |
| --- | --- | --- |
| **Overview** \| **Package** \| Class \| **Tree** \| **Deprecated** \| **Index** \| **Help** \| **Weka's home** | |  |
| **PREV PACKAGE**   **NEXT PACKAGE** | **FRAMES**    **NO FRAMES**     **All Classes** |  |


---

## Package weka.datagenerators.classifiers.classification

| **Class Summary** | |
| --- | --- |
| **Agrawal** | Generates a people database and is based on the paper by Agrawal et al.:  R. |
| **BayesNet** | Generates random instances based on a Bayes network. |
| **LED24** | This generator produces data for a display with 7 LEDs. |
| **RandomRBF** | RandomRBF data is generated by first creating a random set of centers for each class. |
| **RDG1** | A data generator that produces data randomly by producing a decision list.  The decision list consists of rules.  Instances are generated randomly one by one. |

---


|  |  |  |
| --- | --- | --- |
| **Overview** \| **Package** \| Class \| **Tree** \| **Deprecated** \| **Index** \| **Help** \| **Weka's home** | |  |
| **PREV PACKAGE**   **NEXT PACKAGE** | **FRAMES**    **NO FRAMES**     **All Classes** |  |


---
